# Prevalence and clinical aspects of os trigonum: a meta-analysis

**DOI:** 10.1007/s12565-024-00811-4

**Published:** 2024-11-26

**Authors:** Maciej Preinl, Aleksander Osiowski, Kacper Stolarz, Maksymilian Osiowski, Dominik Taterra

**Affiliations:** 1 Faculty of Medicine, Jagiellonian University Medical College, Sw. Anny 12, 31-008 Krakow, Poland; 2 Department of Orthopedics and Rehabilitation, Jagiellonian University Medical College, Balzera 15, 34-500, Zakopane, Poland; 3 Ortho and Spine Research Group, Zakopane, Poland

**Keywords:** Accessory ossicle, Anatomy, Foot and ankle anatomical variation, Os trigonum, Posterior ankle impingement syndrome

## Abstract

Os trigonum (OT) is one of the most common accessory ossicles in the foot and ankle region. It is believed that OT was first described by Rosenmuller in 1804, but von Bardeleben is the one who coined the nomenclature for this anatomical variation in 1883. The literature indicates that OT is extremely variable in prevalence, with reported rates ranging from 1.7% to 32.5%. In clinical practice, OT is a considerable factor that may predispose patients to posterior ankle impingement syndrome (PAIS). The aim of this study was to provide comprehensive data on the prevalence and anatomical characteristics of OT. We conducted a comprehensive search of the main electronic databases to find the relevant research. Information concerning geographical origin, prevalence, gender distribution, imaging modality, and morphometry of OT was extracted. The PRISMA guidelines were strictly followed throughout the study. The reliability of the included studies was assessed using the AQUA tool. A total of 41 studies (n = 36,612 feet) were included in this meta-analysis. This meta-analysis showed that OT was present in 9.0% (95% CI: 7.4–10.8) of the feet, and was present bilaterally in 32.7% (95% CI: 23.3–43.7) of cases. Regarding the types of imaging modality used in the evaluation of OT by the included studies, prevalence assessed by X-rays was 8.2% (95% CI: 6.8–9.9), MRI was 24.2% (95% CI: 14.6–37.3), CT was 21.0% (95% CI: 12.8–32.5), and cadaveric dissection was 5.0% (95% CI: 3.4–7.4). OT was most commonly found in East Asia (11.0%, 95% CI: 7.1–16.8). Two studies provided enough data to calculate the odds ratio linking PAIS with OT. The analysis showed that patients with PAIS are almost 16 times more likely to have OT compared to those without PAIS (OR = 15.98, 95% CI = 0.255–1002.8). OT is a very common accessory ossicle and is present in nearly one in 10 feet. The highest prevalence is noted in the East Asian population. MRI and CT show the highest prevalence of OT. It is important to keep in mind the possible presence of OT when a patient is diagnosed with PAIS.

**Supplementary Information:**

The online version contains supplementary material available at 10.1007/s12565-024-00811-4.

## Introduction

**Fig. 1:**
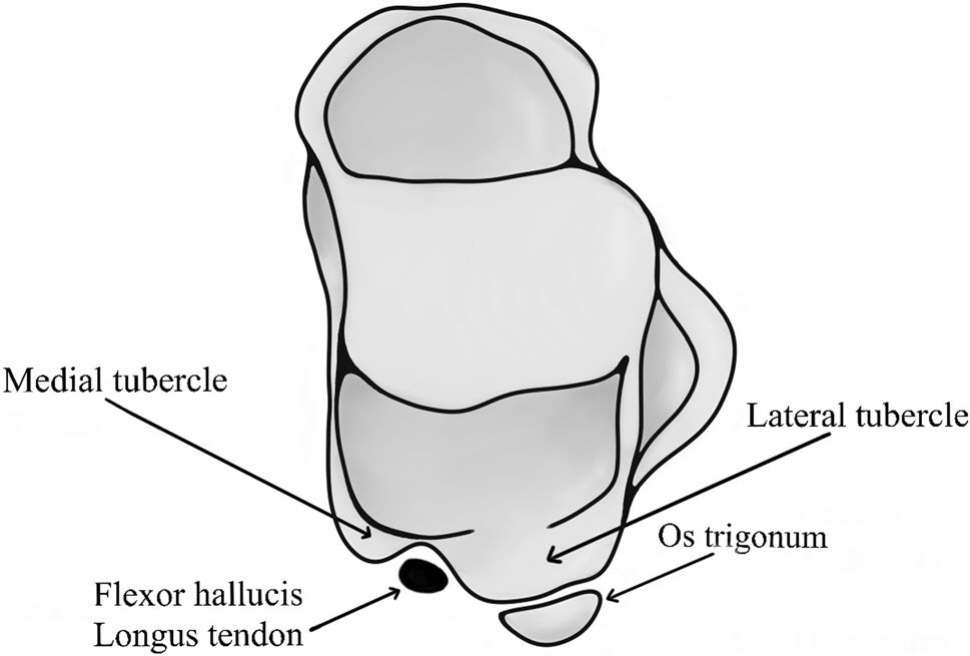
Illustrative diagram of os trigonum in relation to talus

Among many different accessory ossicles, os trigonum (OT) is certainly one of the most common findings in the foot and ankle region (Keles-Celik et al. 2017). According to the literature, the prevalence of OT varies widely, and its reported rates range from 1.7% to 32.5% (Mann and Owsley 1990; Yilmaz and Baykara 2008). Rosenmuller is believed to have been the first to describe it in 1804 (Rosenmuller 1804), but the first to introduce the term “os trigonum” was von Bardeleben in 1883 (Bardeleben 1885). The posterior process of the talus is composed of two tubercles, the medial tubercle and the lateral tubercle, which are additionally separated by the tunnel for the flexor hallucis longus tendon (Matsui 1964). In childhood, between the ages of 8 and 11 years, an additional ossification center appears behind the posterior process of the talus (Lawson 1994). If the new ossicle forms as a distinct bone next to the posterior process's lateral tubercle without a fusion, it is subsequently named OT **(**Fig. 1**)**. However, if the new ossicle fuses with the lateral tubercle, it forms an enlarged and elongated process, which in the literature has been described as the “trigonal process” (Rosenmuller 1804) or “Stieda’s process” (Sopov et al. 2000).

**Fig. 2:**
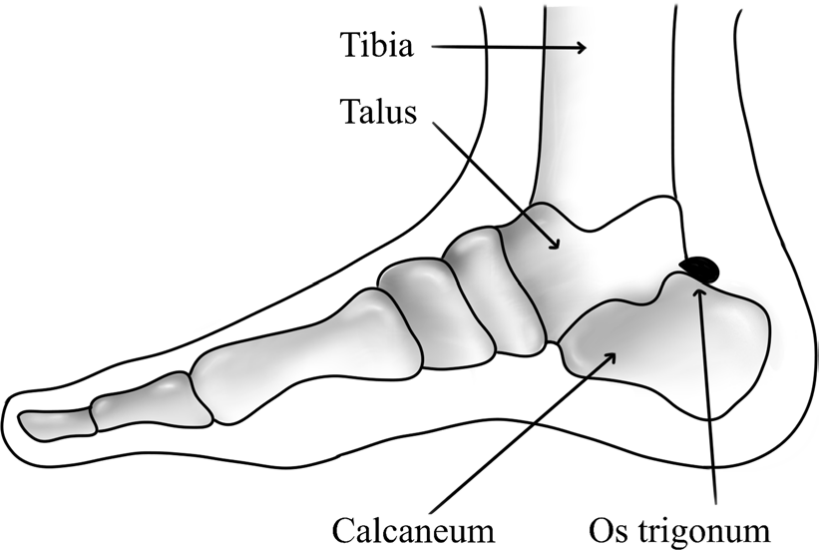
Medial view of posterior ankle with the presence of os trigonum

Posterior ankle impingement syndrome (PAIS) is a clinical condition that can be described as a presentation of sharp pain in the posterior aspect of the ankle, usually arising with a forced plantar flexion. Variations of the posterior aspect of the talus, like the Stieda process and the OT, are the most common causes of PAIS (Tsuruta et al. 1968) **(**Fig. 2**)**. Zwiers et al. 2017 showed that patients diagnosed with PAIS are even more likely to possess an OT. Athletes such as ballet dancers and football players are thought to be more vulnerable to developing this condition due to the nature of their sport, which involves repetitive ankle plantar flexion (Hamilton 1988; Palmer et al. 2020). Treatment of PAIS involves the surgical removal of the excessive bony material if an adequate non-operative approach fails. Options for surgery include arthroscopic or open resection of the accessory ossicle (Vasiljevic 2010).

The reviewed literature contained insufficient data describing the prevalence of OT. Therefore, the aim of the current study was to unify the data on the prevalence of OT by performing a meta-analysis of 41 studies and over 36.000 feet with multiple subgroups analyses, including gender, imaging modality, and geographical region.

## Methods

### Search strategy

Up to March 2024, major electronic databases (Pubmed and Embase) were searched for research on the OT. The following search terms were employed: os trigonum OR ossa trigona OR trigonal process OR stieda process OR posterior ankle impingement. The original language and the publishing date were not taken into consideration as exclusion criteria. Further reference checking was done in order to locate other possibly acceptable publications that were previously overlooked while obtaining the full texts. This study adhered closely to the preferred reporting items for systematic reviews and meta-analyses (PRISMA) standards (Henry et al. 2016).

### Eligibility assessment

Every possible article was evaluated for eligibility by two separate reviewers. Included were all studies that provided comprehensive data on the OT prevalence. Studies that (1) offered inadequate or incomplete data, or data that were hard to extract; (2) were conducted on animals; and (3) were published as case reports and case series, letters to the editor, review articles, and conference abstracts were not included in this meta-analysis. Several investigations were conducted on diverse patient groups, such as football players, ballet dancers, postmortem examination studies, and others. As a result, a consensus was reached to include research that reported OT existence in these various cases in the meta-analysis. When the writers were not proficient in the publication's language, assistance from medical practitioners who were fluent in both languages was sought. Every reviewer took part in the evaluation until an agreement was reached in cases where there was disagreement on inclusion in the study.

### Data extraction

Two reviewers independently extracted data from every study that satisfied the inclusion criteria for this meta-analysis. Information was gathered on the sample size, geographic distribution, imaging modality (XRAY, MRI, CT, or dissections), as well as gender distribution, underlying pathology, and anatomical variations of the OT.

### Study end-points

The primary end-point was to determine the prevalence of OT in the general population. The secondary end-points focused on distribution in subgroups based on gender, geographic distribution, and imaging modality.

### Statistical analysis

Statistical analysis was carried out by two reviewers. The authors conducted the analysis using Comprehensive Meta-Analysis Version 4 (Borenstein et al. 2022). The pooled prevalence estimates (PPE) of OT were computed using a random effects model. χ2 and I2 were used to measure the heterogeneity of the included studies. In relation to the I2 statistic, the heterogeneity was found to “may not be significant” at values of 0–40%, “may indicate moderate heterogeneity” at 30–60%, “may indicate substantial heterogeneity” at 50–90%, and “may represent considerable heterogeneity” at 75–100%. Significant heterogeneity between the examined works was indicated by a p value for Cochran's Q of less than 0.10.

Subgroup analysis was carried out, taking into account the patient's gender distribution, the study type, and the study's geographic origin. Subgroup data were extracted from the analyzed studies based on their availability. Confidence intervals (CIs) were used to identify significant differences between the subgroups under analysis. If the two rates' confidence intervals overlapped, it meant that there was no statistically significant difference between them. To examine potential causes of heterogeneity in greater detail, sensitivity was assessed using a leave-one-out analysis.

### The difference between the feet and patient prevalence

Frequency per foot and frequency per patient must be distinguished in order to properly characterize prevalence statistics as described by Benjamin M. Ochs in 2021 (Ochs 2021). When OT is present bilaterally in a patient, both frequencies per foot and per patient are equal, but when OT is only presented unilaterally, the foot-prevalence is half in comparison to the patient prevalence. In the studies analyzing prevalence of OT per patient, if all the necessary data with a separate distinction concerning cases with bilateral ossicles was present, the prevalence per foot was recounted.

### Quality assessment

The AQUA tool (Henry et al. 2017) was used by the reviewers to evaluate the quality and reliability of the included studies. In brief, the tool was devised in order to probe for potential bias (Henry et al. 2017). Five domains were evaluated in the analysis: (1) objective(s) and subject, (2) study design, (3) methodology characterization, (4) descriptive anatomy, and (5) reporting of results; and each domain was categorized as either “low,” “high,” or “unclear” risk of bias (Henry et al. 2017). A decision was made that a “no” answer in whichever signaling question within each of the categories arbitrated the domain to be of “high” risk of bias, whereas all answers “yes” suggested that it presented a “low” risk of bias. The “unclear” option was chosen when the study with incoherent data did not permit clear scrutiny (Henry et al. 2017).

## Results

### Study identification and characteristics of the included studies

**Fig. 3:**
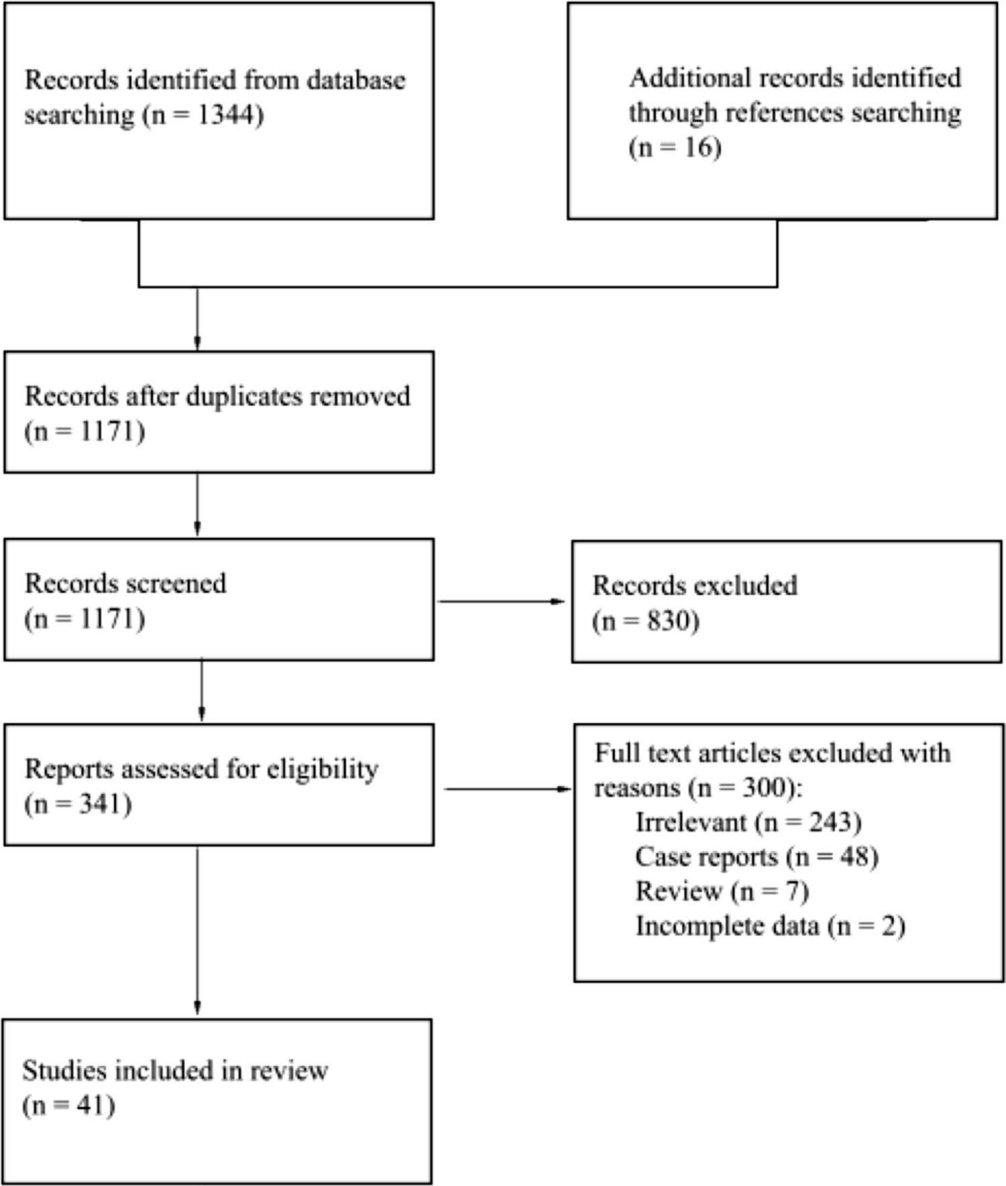
Flowchart of the meta-analysis

The characteristics of the study identification process are presented in Fig. 3**.** The initial database search showed 1344 entries, and after removing the duplicates, the number of entries was 1155. Sixteen additional studies were identified during the reference search. There were 1171 articles found in the preliminary search that could potentially meet inclusion criteria. After the initial screening of abstracts and titles, 830 records were excluded. A total of 341 articles were deeply analyzed in full text, and data were extracted. After further exclusion due to various reasons (case reports, case series, reviews, lack of full-text articles, irrelevant or insufficient data, different research performed on the same patient group), a total of 41 studies (Bizarro 1921; Burman 1931; Candan et al. 2022; Cankaya and Ogul 2020; Capecchi 1962; Cicek and Bankaoglu 2020; Cilli and Akçaoğlu 2005; Coskun et al. 2009; Fu 2019; Geist 1915; Grant 1962; Heimerzheim 1925; Holle 1938; Kalbouneh et al. 2019, 2021; Kalbouneh 2021; Khan 2017; Kır 2011; Kleinberg 1917; Knapik et al. 2017; Koo et al. 2017; Lee et al. 2020; Leimbach 1937; Mann and Owsley 1990; Matsui 1964; Nikaido 1959; Ochs 2021; Özer and Yıldırım 2019; Peace et al. 2004; Sarrafian 1983; Scheuermann et al. 2018; Schönekeß 1935; Sewell 1904; Shands and Durham 1931; Sopov et al. 2000; Stolarz et al. 2024; Tawfik et al. 2020; Thomson 1890; Trolle 1948; Tsuruta et al. 1968; Yasui et al. 2016; Yilmaz and Baykara 2008) were finally included in this meta-analysis. The characteristics of the included studies are presented in Table 1.

**Table 1:** Studies included in the meta-analysis

Study ID	Origin of the study	Type of study	Total number of ankles	OT in feet
Bizarro 1921	England	X-ray	100	7
Burman 1931	USA	X-ray	1000	64
Candan 2022	Turkey	X-ray	1651	163
Cankaya and Ogul 2020	Turkey	MRI	129	18
Capecchi 1962	Italy	X-ray	2155	296
Cicek and Bankaoglu 2020	Turkey	X-ray	1088	101
Cilli and Akçaoğlu 2005	Turkey	X-ray	464	20
Coskun et al. 2009	Turkey	X-ray	1968	32
Fu 2019	China	CT	1011	275
Geist 1915	USA	X-ray	200	7
Grant 1962	USA	Cadaveric	558	43
Heimerzheim 1925	Germany	X-ray	1800	78
Holle 1938	Germany	X-ray	1000	188
Kalbouneh 2019	Jordan	X-ray	798	163
Kalbouneh 2021	Jordan	X-ray	1000	154
Kalbouneh 2021	Jordan	CT	1478	303
Khan 2017	Canada	MRI	172	69
Kır 2011	Turkey	X-ray	554	21
Kleinberg 1917	USA	X-ray	350	19
Knapik 2017	USA	X-ray	261	37
Koo 2017	Korea	X-ray	44	14
Lee 2020	Korea	X-ray	532	31
Leimbach 1937	Germany	X-ray	500	23
Mann and Owsley 1990	USA	Cadaveric	813	14
Matsui 1964	Japan	X-ray	213	11
Nikaido 1959	Japan	X-ray	1315	70
Ochs 2021	Germany	X-ray	1820	185
Özer 2019	Turkey	MRI	333	72
Pfitzner 1896	Germany	Cadaveric	841	51
Scheuermann 2018	Chile	X-ray	410	28
Schönekeß 1935	Germany	X-ray	1324	108
Sewell 1904	Egypt	Cadaveric	1006	110
Shands 1931	USA	X-ray	1101	61
Sopov 2000	Israel	X-ray	200	31
Stieda 1899	Germany	Cadaveric	305	18
Suzuki 1957	Japan	X-ray	701	68
Thomson 1890	England	Cadaveric	876	12
Tsuruta 1968	Japan	X-ray	1449	148
Tsuruta 1981	Japan	X-ray	3460	438
Yilmaz 2008	Turkey	Cadaveric	376	28
Zwiers 2017	Netherlands	CT	1256	294

*OT* os trigonum

### Quality assessment

The majority of the studies included in this meta-analysis, evaluated by the AQUA tool, revealed a “low” risk of bias in each of the five domains. Some studies were categorized as having an “unclear” risk of bias, owing mainly to following considerably different patterns of reporting the data (studies published in the late 1800s and early 1900s). Only a small percentage of studies had been categorized as having a “high” risk of bias in every domain (Supplementary 1).

### General prevalence of OT

**Fig. 4:**
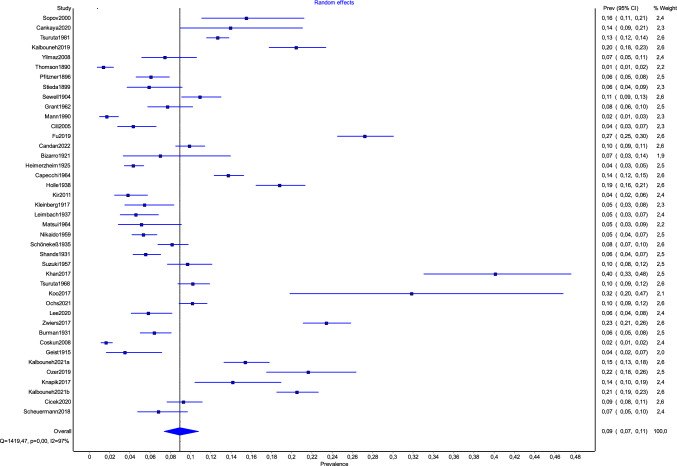
Forest plot presenting general prevalence of os trigonum

Overall, the number of studies included in this analysis was 41 (36 612 feet, of which 3 873 had OT). The PPE of OT was 9.0% (95% CI: 7.4–10.8) (Fig. 4). The results of general prevalence of OT are presented in Table 2.

**Table 2:** Meta-analysis presenting general prevalence of os trigonum with gender distribution

Subgroup	Number of studies (number of feet)	Prevalence of OT: % (95% CI)	I^2^: %	Cochran’s Q, p value
General	41 (36 612)	9.0 (7.4–10.8)	97.192	< 0.001
Male	13 (8 266)	12.7 (10.1–15.8)	97.20	< 0.001
Female	12 (7 635)	12.4 (9.1–16.6)	97.15	< 0.001

*OT* os trigonum

### Gender prevalence

In total, the number of studies included in this analysis was 13 (7 635 female and 8 266 male feet). Pooled prevalence was estimated to be 12.4% (95% CI: 9.1–16.6) for females and 12.7% (95% CI: 10.1–15.8) for males. There was no statistically significant difference between the gender-based groups. (Table 2).

### Prevalence based on types of studies

From a total of 36,612 feet, 27,458 were examined by X-ray, 4775 were assessed during cadaveric dissections, 3745 were analyzed with computed tomography (CT), and 634 were analyzed with magnetic resonance imaging (MRI). In the X-ray group, 2 566 feet had OT (6.6% (95% CI: 4.3–9.9)), 276 feet had OT in cadaver dissections (5.0% (95% CI: 3.4–7.4)), 872 feet examined on CT (21% (95% CI: 12.8–32.5)), and 159 feet examined on MRI (24.2% (95% CI: 14.6–37.3)). Modalities characterized by higher quality (MRI and CT) present a statistically significant difference between them and other types of examinations **(**Table 3**).**

**Table 3:** Meta-analysis presenting comparison of prevalences of os trigonum based on different types of studies

Type of imaging modality proceeded	Number of studies (number of feet)	Prevalence of OT: % (95% CI)	I^2^: %	Cochran’s Q, p value
Cadaveric dissection	7 (4 775)	5.0 (3.4–7.4)	93.23	< 0.001
X-ray	28 (27 458)	8.2 (6.8–9.9)	95.69	< 0.001
CT	3 (872)	21.0 (12.8–32.5)	95.04	< 0.001
MRI	3 (634)	24.2 (14.6–37.3)	93.28	< 0.001

*OT* os trigonum

### Geographical distribution

When OT’s global distribution was examined, 35,196 feet were analyzed in the statistics from four regions of the world. The highest prevalence of OT was noted in East Asia (11.0%, 95% CI: 7.1–16.8). The second highest prevalence was observed in the Middle East (9.9% (95% CI: 6.9–14.1)), then in Europe (7.5%, 95% CI: 5.0–10.9), and in North America (7.5%, 95% CI: 4.7–11.7) **(**Table 4**)**.

**Table 4:** Meta-analysis comparing os trigonum prevalence between ethnicity-based groups

Geographic region	Number of studies (number of feet)	Prevalence of OT % (95% CI)	I^2^: %	Cochran’s Q, p value
East Asia	8 (8 725)	11.0 (7.1–16.8)	97.61	< 0.001
Middle East	12 (10 039)	9.9 (6.9–14.1)	97.16	< 0.001
Europe	11 (11 977)	7.5 (5.0–10.9)	96.71	< 0.001
North America	8 (4 455)	7.5 (4.7–11.7)	97.06	< 0.001

*OT* os trigonum

### Laterality of OT

Total number of studies that included information about laterality of OT and were included in this analysis was 7, carried out on 2 644 patients, in which OT was identified in 314 cases. From this number, 194 patients had OT unilaterally (67.3% (95% CI: 56.3–76.7)) and 120 bilaterally (32.7% (95% CI: 23.3–43.7)) (Table 5).

**Table 5:** Meta-analysis comparing laterality of os trigonum

Type of laterality	Prevalence of OT % (95% CI)	I^2^: %	Cochran’s Q, p value
Unilateral	67.3 (56.3–76.7)	49.47	< 0.001
Bilateral	32.7 (23.3–43.7)	49.47	< 0.001

*OT* os trigonum

### Relationship between PAIS and OT

Only two studies (Özer and Yıldırım 2019; Zwiers 2017)provided sufficient data to calculate the odds ratio linking the occurrence of PAIS with the presence of OT. The statistical analysis revealed that patients suffering from PAIS are almost 16 times more likely to have the OT when compared to patients without PAIS (OR = 15.98, 95% CI = 0.255–1002.8).

## Discussion

Among accessory ossicles in the foot and ankle region, presence of the OT is a clinically important and common anatomical variation. It is located near the posterior aspect of the talus and adjacent to the posterior talofibular ligament. In terms of development, OT originates from an ossification center that appears during childhood. In some individuals, this center does not fuse with the talus, resulting in the formation of the OT. When a patient appears with discomfort or conflicts in the posterior ankle region, all clinicians should be aware of the presence of OT as it can help with a more accurate diagnosis.

The objective of this research was to systematically examine the literature and provide the most up-to-date information on the prevalence, anatomy, and morphology of OT. The study also examined its relationship with posterior ankle impingement syndrome. This is the first meta-analysis of the prevalence of OT, with sub-analyses of geography and its correlation to PAIS. The prevalence of the OT in previous research was determined in two ways: the prevalence in patients and the prevalence per foot. If the author stated that each patient had both feet analyzed and reported data about the laterality of OT, we applied proper calculations to assess the prevalence per foot. Due to the impossibility of direct conversion from prevalence per patient to prevalence per foot, two studies were excluded from our analysis (Longo 2013; Tsuruta et al. 1981).

### Anatomical characteristics

The most important finding of this study was that the prevalence of OT in the general population was 9%. This meta-analysis showed that OT is one of the most common accessory ossicles in the foot, which correlates with the vast majority of authors, including the biggest studies considering this topic (Coskun et al. 2009; Kalbouneh et al. 2021; Ochs 2021; Tsuruta et al. 1968).

Pooled prevalence estimates of OT were found to be similar in women and men (12.4% and 12.7%, respectively). Analyses in the current study failed to find statistically significant differences between the gender-based subgroup. These results also show that in almost every third patient with OT, the ossicle is presented bilaterally.

### Developmental factors

Zwiers et al. 2017 obtained a significantly higher prevalence of OT of 32.5% in patients affected by posterior ankle impingement syndrome. Peace et al. 2004 determined the prevalence of OT in the group of 25 ballerinas to be over 30%. Such high OT may be due to the specificity of sports performed by their participants at a young age with frequent and loaded maintenance of plantar flection, therefore at the age when the ossification process still occurs (Palmer et al. 2020). Lawson (Lawson 1994) showed that the onset of ossicle formation in females happens between the ages of 8 and 10, whereas in men it appears between the ages of 10 and 13 years. In their 1000-ft study, Burman and Lapidus (Burman 1931) determined the prevalence of the OT at 49.3%. However, they took into account both free and fused (process still attached to the talus) ossicles and only 64 of them were isolated, and that is the sample used in the statistics, which is compatible with other authors (Kalbouneh et al. 2019; Yilmaz and Baykara 2008). Trolle 1948 in a study performed on embryos reported that no OT was found during his research. However, Trolle 1948 reported the presence of other accessory ossicles (accessory navicular bone, os peroneum, os metatarseum), which suggests that that ossification and formation of accessory bones in the foot differ over time and that some of them may already exist in the prenatal period.

### Regional and ethnic differences in prevalence

The prevalence of OT increases with age and differs for particular age groups (Capecchi 1962). Due to the diverse studies included in this meta-analysis and the lack of specific age ranges among most authors, the current study could not demonstrate such a relationship. Differences in the presence of the OT may appear not only in different age ranges but also in different geographical regions. Mann and Owsley (Mann and Owsley 1990) did not notice any OT in the group of native Americans. Zwiers (2017) described patients from the Caribbean, Surinamese, or Central African descent, which showed a lower prevalence of OT. Our study proved that the highest prevalence of OT was in the Asian population, which might indicate a genetic predisposition in comparison to other populations. Although different geographical regions showed various results, the results failed to prove to be statistically significant.

Despite knowing countries of origin of included studies, information about the ethnicity of the patients included in each study remains unknown. Countries with high ethnic diversity (e.g., the USA) introduce bias in our analysis. Another limitation is the lack of full information on the history of the examined patients (about their work, amateur or professional sports history, etc.), which, as previously mentioned, may significantly affect the prevalence of OT.

### Identification of os trigonum

The results of this study indicate that the accuracy of the identification of OT depends strongly on the imaging modality. X-ray examination is the most frequently used test, but its accuracy for bone structure depends on the quality of the images. The highest prevalence of OT occurred with the use of MRI and CT due to their high sensitivity and specificity. This may indicate that the actual prevalence of OT is higher, and the results of previous studies may be underestimated because of the effectiveness of the imaging modality assessed in the study.

### Symptomatic association with posterior ankle impingement syndrome

The most frequent problem brought on by OT is PAIS, which is caused by terminal plantar flexion compressing tissues posterior to the tibiotalar and talocalcaneal articulations (Lavery et al. 2016). Since some sports involve persistent hyperplantar flexion, ballet dancers, basketball players, and soccer players are more likely to develop this condition, especially when it occurs at the age of the ossification process, which makes them more likely to experience pain in the ankle area ((Hamilton 1988; Miller 2002)). That phenomenon causes them to consult a specialist more often, which results in more frequent diagnoses of OT than in the average population (Nault et al. 2014). Whether there is an association of PAIS with OT remains controversial. Our finding of the relationship between PAIS and OT is subject to some limitations. Those involve a small number of studies (2) and patients (961) included in the analysis, as well as the major discrepancy in reports between the only 2 studies involved in the analysis (OR in Zwiers 2017 study = 1.99; OR in Ozer2019 study = 136). The result of our analysis did not reach statistical significance. In order to clearly establish the relation between the occurrence of PAIS and the presence of OT, further investigation is necessary. Nonetheless, it is strongly recommended that in every patient presenting with the symptoms of PAIS, the presence of OT should be evaluated as a possible causative factor of this condition.

### Treatment approaches

Treatment of this condition might be divided into two main approaches: nonsurgical treatment, which is advisable as a first-line treatment and involves the use of NSAIDs, ice, and shoe modifications, including heel lift orthoses (Lavery et al. 2016). The second approach is surgery, recommended for enduring symptoms that have not improved with non-operative care, interfered with everyday activities or athletic performance, and are consistent with imaging and physical examination results (Lavery et al. 2016).

### Classification of os trigonum

Information on the classification of the OT into subtypes is rarely provided by authors, and only a fraction of authors give the prevalence along with the 3 main types ((Candan et al. 2022; Fu 2019)). There is no clear position on which subtype is the most common. In her research, Candan (Candan et al. 2022) determined that type II was the most prevalent subtype, although Fu et al. (Fu 2019) determined that type III was the most prevalent. The reason for such a situation may be due to the fact that the first classification of OT, regarding its relation to the posterior aspect of the talus, was conducted by Zwiers et al. 2017. However, Fu et al. (Fu 2019) in 2019 were the first to classify OT into three basic categories depending on the manner of relation to talus: Type I is a single piece of bone that is not related to the talus; Type II is a hyaline cartilage layer that is connected to the posterior talar process; and Type III is Stieda's process. Divisions of other bones (e.g., accessory navicular bone) were introduced earlier (Geist 1915; Stieda 1899), are more standardized, and are reported by more authors.

### Study limitations

Our investigation was limited because of the substantial degree of variation within the included research. The significant degree of heterogeneity persisted throughout the trial, despite our attempts to employ subgroup analysis to look into its source. In studies reporting symptomatic sample of patients (e.g., in suspicion of PAIS) who were assessed with CT and/or MRI, it can be speculated that the reported prevalence of OT may be higher compared to the general, asymptomatic population and thus, may partially explain the observed heterogeneity. The study protocol was not registered before this systematic review and meta-analysis, which is another disadvantage. Although it is recommended, the global survey revealed that it is not a widely used strategy (Tawfik et al. 2020). The lack of studies in Australia/Oceania/South America may limit generalizability of the findings.

Our study is not the first to analyze the prevalence of OT in the general population. However, in comparison to the previously published meta-analysis by Ráfare et al. 2024, in which only 18 studies were included, our analysis consists of 41 studies (36,612 feet), which results from a more profound and thorough literature search and full-text extraction process and, moreover, reflects more accurate and precise results. In Table 1, “Overall prevalence of the os trigonum extracted from the studies included in the present meta-analysis” reference number 33—the number of patients (438) was incorrectly assumed as the number of feet, which should have been 876. The author (Thomson 1890)clearly emphasizes that there were 438 specimens analyzed in the study. This detail was observed by Benjamin M. Ochs (Ochs 2021). In addition, in contrast to the previous study (Ráfare et al. 2024), our meta-analysis is the first to present multiple subgroup analyses focused on geographical distribution and laterality. As another advantage, our study is the first to try to investigate and assess a correlation between PAIS and the existence of OT, depicted as an odds ratio analysis.

## Conclusions

This study showed that OT is a very common accessory bone that occurs one in 10 feet. In every third patient with OT, the ossicle is present bilaterally. Populations living in Asia and the Middle East have the highest prevalence of OT. Imaging tests such as MRI or CT have a greater chance of locating OT than X-rays. When a patient presents with posterior ankle impingement syndrome, OT should be taken into consideration as a possible cause.

## Supplementary Information

Below is the link to the electronic supplementary material.

Supplementary file1 (PDF 98 kb)

## Data Availability

The original publications provided the data used in the manuscript. Upon request, the corresponding author can supply the entire set of data used in this analysis.
