# Supplementary material for: Prevalence and clinical aspects of os trigonum: a meta-analysis
Source: Anat Sci Int. 2024 Nov 26;100(3):287–97. doi: 10.1007/s12565-024-00811-4 (PMC12043738; doi:10.1007/s12565-024-00811-4)
Supplement: Supplementary file 1 — Supplementary file1 (PDF 98 kb) [file 12565_2024_811_MOESM1_ESM.pdf]

Prevalence and Clinical Aspects of Os trigonum: A meta-analysis

Maciej Preinl[1,3], Aleksander Osiowski[1,3], Kacper Stolarz[1,3], Maksymilian Osiowski[1,3], Dominik Taterra[2,3]

Affiliations

1. Faculty of Medicine, Jagiellonian University Medical College, sw. Anny 12, 31-008 Krakow, Poland

2. Department of Orthopedics, Jagiellonian University Medical College, Balzera 15, 34-500 Zakopane, Poland

3. Ortho and Spine Research Group, Zakopane, Poland

Corresponding author:

Dominik Taterra, MD

dominik.taterra@gmail.com

Department of Orthopedics and Rehabilitation

Jagiellonian University Medical College

Zakopane, Poland

RISK OF BIAS				
Study	OBJECTIVE(S) AND STUDY CHARACTERISTICS	STUDY DESIGN	METHODOLOGY CHARACTERIZATION	DESCRIPTIVE ANATOMY
Sopov2000	UNCLEAR	HIGH	LOW	UNCLEAR
Cankaya2021	LOW	LOW	LOW	LOW
Tsuruta1981	LOW	LOW	LOW	LOW
Kalbouneh2019	LOW	LOW	LOW	LOW
Yilmaz2008	LOW	LOW	LOW	LOW
Zwiers2017	LOW	LOW	LOW	LOW
Thomson1890	UNCLEAR	UNCLEAR	UNCLEAR	UNCLEAR
Pfitzner1896	UNCLEAR	UNCLEAR	UNCLEAR	UNCLEAR
Stieda1899	UNCLEAR	UNCLEAR	UNCLEAR	UNCLEAR
Sewell1904	UNCLEAR	UNCLEAR	UNCLEAR	UNCLEAR
Burman1931	UNCLEAR	UNCLEAR	UNCLEAR	UNCLEAR
Grant1962	LOW	HIGH	HIGH	LOW
Mann1990	HIGH	LOW	LOW	HIGH
Cilli2005	LOW	LOW	LOW	LOW
Coskun2008	LOW	LOW	LOW	LOW
Fu2019	LOW	LOW	LOW	LOW
Candan2022	LOW	LOW	LOW	LOW
Bizarro1921	UNCLEAR	UNCLEAR	UNCLEAR	UNCLEAR
Geist1915	UNCLEAR	UNCLEAR	UNCLEAR	UNCLEAR
Heimerzheim1925	UNCLEAR	UNCLEAR	UNCLEAR	UNCLEAR
Capecchi1964	HIGH	HIGH	HIGH	HIGH
Holle1938	UNCLEAR	UNCLEAR	UNCLEAR	UNCLEAR
Kir2011	LOW	LOW	LOW	LOW
Kleinberg1917	UNCLEAR	UNCLEAR	UNCLEAR	UNCLEAR
Leimbach1937	UNCLEAR	UNCLEAR	UNCLEAR	UNCLEAR
Matsui1964	LOW	LOW	HIGH	HIGH
Nikaido1959	LOW	LOW	HIGH	HIGH
Schönekeß1935	UNCLEAR	UNCLEAR	UNCLEAR	UNCLEAR
Shands1931	UNCLEAR	UNCLEAR	UNCLEAR	UNCLEAR
Suzuki1957	HIGH	HIGH	LOW	LOW
Khan2017	LOW	LOW	LOW	LOW
Kalbouneh2021a	LOW	LOW	LOW	LOW
Ozer2019	LOW	HIGH	LOW	LOW
Tsuruta1968	LOW	LOW	LOW	LOW
Lee2020	LOW	LOW	LOW	LOW
Koo2017	LOW	HIGH	LOW	HIGH
Ochs2021	LOW	LOW	LOW	LOW
Scheuermann2018	LOW	LOW	LOW	LOW
Cicek2020	LOW	LOW	LOW	LOW
Kalbouneh2021b	LOW	LOW	LOW	LOW
Knapik2017	LOW	LOW	LOW	LOW
